# Long-range control of gene expression via RNA-directed DNA methylation

**DOI:** 10.1371/journal.pgen.1006749

**Published:** 2017-05-05

**Authors:** M. Jordan Rowley, M. Hafiz Rothi, Gudrun Böhmdorfer, Jan Kuciński, Andrzej T. Wierzbicki

**Affiliations:** University of Michigan, Department of Molecular, Cellular, and Developmental Biology, Ann Arbor, Michigan, United States of America; Gregor Mendel Institute of Molecular Plant Biology, AUSTRIA

## Abstract

RNA-mediated transcriptional silencing, in plants known as RNA-directed DNA methylation (RdDM), is a conserved process where small interfering RNA (siRNA) and long non-coding RNA (lncRNA) help establish repressive chromatin modifications. This process represses transposons and affects the expression of protein-coding genes. We found that in *Arabidopsis thaliana* AGO4 binding sites are often located distant from genes differentially expressed in *ago4*. Using Hi-C to compare interactions between genotypes, we show that RdDM-targeted loci have the potential to engage in chromosomal interactions, but these interactions are inhibited in wild-type conditions. In mutants defective in RdDM, the frequency of chromosomal interactions at RdDM targets is increased. This includes increased frequency of interactions between Pol V methylated sites and distal genes that are repressed by RdDM. We propose a model, where RdDM prevents the formation of chromosomal interactions between genes and their distant regulatory elements.

## Author summary

Repetitive elements, such as transposons, comprise a large portion of the genome that is kept silent to maintain genome integrity. Transposon silencing is a canonical role of RNA-directed DNA methylation (RdDM) where small interfering RNA (siRNA) and long non-coding RNA (lncRNA) work together to place repressive chromatin marks. In addition to its role in controlling repetitive sequences, RdDM contributes to the regulation of gene expression yet molecular mechanisms utilized by RdDM to control gene expression remain mostly unknown. We show that the repression of gene expression by RdDM may involve preventing interactions between distant chromosomal regions.

## Introduction

Transcriptional gene silencing (TGS) is an important pathway for transposon repression throughout Eukarya. This process involves the establishment of repressive histone modifications and *de novo* DNA methylation under the guidance of non-coding RNA [1,2]. This pathway’s activity is prominent in the germline of Metazoans in establishing *de novo* DNA methylation that can be afterwards maintained [1]. In the model flowering plant species *Arabidopsis thaliana* TGS, also known as RNA-directed DNA methylation (RdDM), occurs throughout development in order to consistently establish and maintain repressive chromatin marks.

RdDM in *Arabidopsis* uses two specialized RNA polymerases, RNA polymerase IV (Pol IV) and RNA polymerase V (Pol V) to direct repressive chromatin modifications [3]. While Pol IV is believed to produce siRNA precursors, Pol V has been proposed to produce lncRNA scaffolds which help guide proteins to chromatin [3–6]. ARGONAUTE 4 (AGO4) is one of those proteins and its interactions with siRNA, lncRNA, and Pol V largest subunit allow its recruitment to chromatin [7,8]. Binding of AGO4 and other RNA binding proteins, such as SPT5L and IDN2, is then important for binding and activity of enzymes, which establish repressive chromatin modifications [9–12].

Mechanisms and roles of DNA methylation established by Pol V-associated factors have been studied extensively [13–16]; however, the TGS / RdDM pathway is not limited to DNA methylation. It has been shown to mediate the establishment of repressive posttranslational histone modifications [7,9,17]. It has also been implicated in the control of nucleosome positioning through the recruitment of a putative ATP-dependent chromatin remodeling complex [12]. These broad effects of RdDM on chromatin structure indicate that this pathway is a master regulator of chromatin status at its target loci.

RdDM not only represses transposon activity but also affects the expression levels of protein-coding genes [8,12,18,19]. This may be partially explained by the widespread presence of short transposons upstream of transcription start sites, where RdDM components preferentially localize and establish DNA methylation [8,19,20]. Indeed, AGO4 binds to putative promoters and establishes DNA methylation [8,14], which has been proposed as a mechanism of RdDM-mediated gene expression control [8]. However, the exact manner by which this occurs remains unresolved.

One way that the RdDM pathway could directly control gene expression is through manipulation of the chromatin landscape at promoters. Stabilized nucleosomes, repressive histone modifications, DNA methylation, or occupancy by RdDM proteins could directly influence transcription factor binding and thereby inhibit transcription. In addition to promoters, many eukaryotes control expression using enhancers, which may be distant from the transcriptional start site (TSS) [21–25]. 3D spatial interactions between loci allow distal enhancers to influence transcription long range. The presence of these long range chromosomal interactions is often associated with active histone modifications and/or nucleosome free regions [21,26–29].

The presence and importance of distant enhancers in plant genomes remains mostly unknown. It has been argued that most *Arabidopsis* genes are controlled by proximal regulatory elements located within 3 kb from the transcription start site [30]. However, the role of longer range effects cannot be excluded, especially under non-standard conditions. Therefore, it remains possible that mechanisms controlling chromatin modifications may also affect the formation of long range chromosomal interactions in *Arabidopsis*, which may influence gene expression levels. Analysis of the chromatin interaction landscape around differentially expressed genes may provide an explanation for how RdDM affects the expression of protein coding genes.

We found that AGO4 binding sites are often distal from genes differentially expressed in *ago4*. We proposed and tested the hypothesis that RdDM affects the frequency of long range chromosomal interactions through comparison of Hi-C contacts in Col-0 (wild-type), *nrpe1* (mutant for the large subunit of Pol V), and *ago4*. Our data indicate that RdDM reduces the frequency of long range chromosomal interactions at methylated loci. We also found that genes differentially expressed in *ago4* gain interactions to previously methylated sites when *AGO4* is mutated. These data support a speculative model where RdDM can control the expression of genes over long distances by inhibiting interactions between promoters and their potential distant regulatory elements.

## Results

### Long distance control of gene expression by RdDM

**Fig 1:**
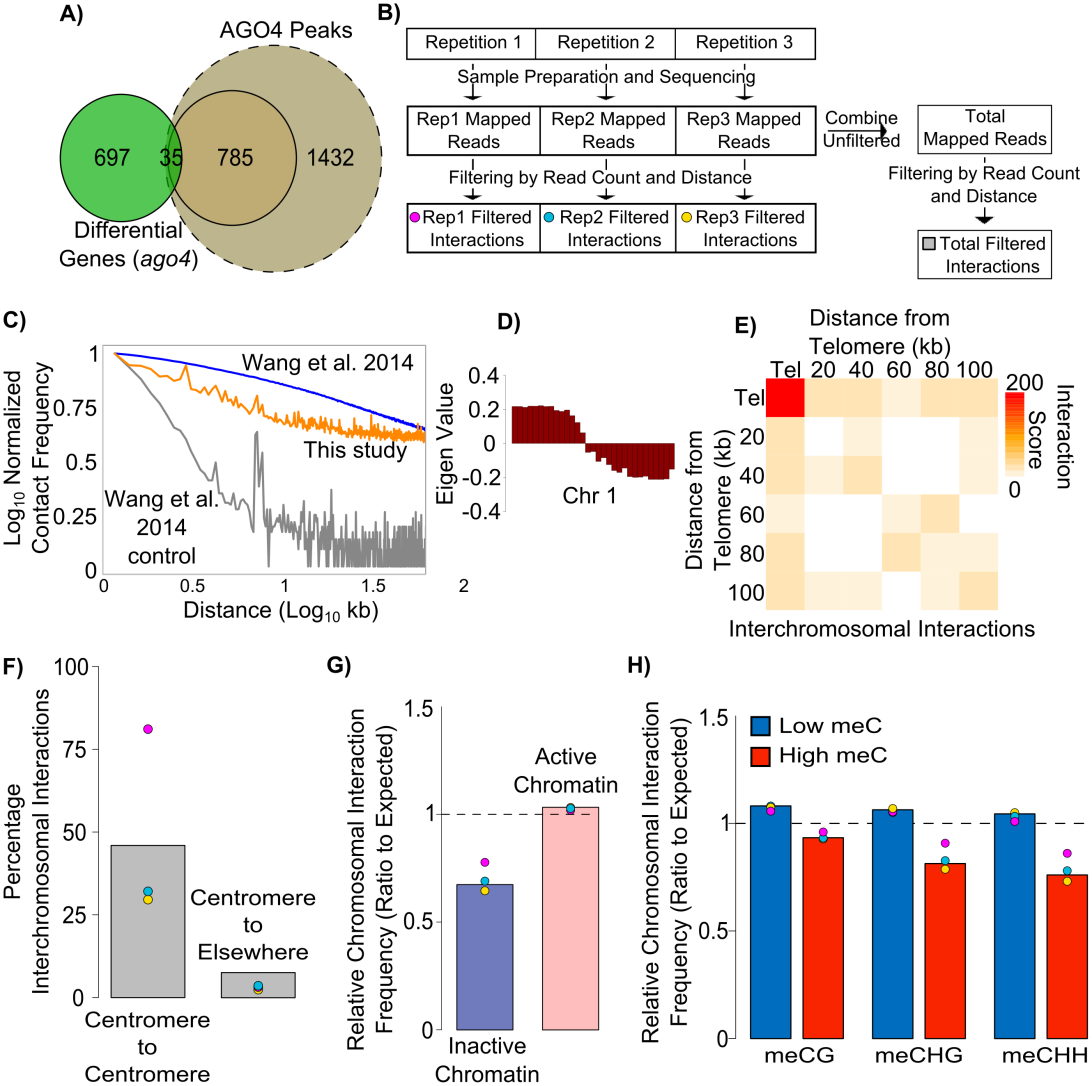
Chromosomal interactions are inhibited in non-centromeric heterochromatin. (A) Overlap between genes with differential expression in the *ago4* mutant (green) and genes with AGO4 peaks defined using high confidence (brown) or lower confidence (light brown) score cutoffs. AGO4 binding to a gene is defined as binding within a region from 2.5 kb upstream of the TSS. (B) Overview of Hi-C data analysis. Hi-C was performed in three biological replicates where each replicate was independently mapped and interactions were scored and filtered by read count and distance. Additionally, total reads were combined and then filtered. Each replicate as well as the combined data are used in the analysis. (C) Decay curves of Col-0 Hi-C compared to previously published *Dpn*II datasets. Reads were aligned to *Dpn*II fragments and kept if >3 *Dpn*II sites apart. These were assigned to 250bp genomic bins and plotted. Col-0 Hi-C presented here (orange) compared to Col-0 Hi-C published by Wang et al. [29] (blue) and their reverse-crosslinked control (grey). (D) Self-association of chromosome arms. Eigenvector plot of contact correlation along chromosome 1. (E) Preferential association of telomeres. Metaplot of inter-chromosomal interactions at telomeres and the surrounding regions in 20 kb bins. (F) Centromeres preferentially interact with other centromeres. Interactions are plotted as a percentage of total high confidence inter-chromosomal interactions. Individual repeats are color-coded as represented in Figure 1B. Gray bars indicates levels found from combining reads before filtering and does not represent the average of three repeats. (G) Non-centromeric inactive chromatin is inhibited from forming chromosomal interactions. Interactions present in Col-0 were plotted if one side was found in either inactive or active non-centromeric chromatin bins defined in S1H Fig. These were plotted as a ratio to the expected (calculated from random bins). Bars represent data from combined replicates while points represent individual replicates color-coded as in Figure 1B. Significance calculated by a t-test of replicates, p≤0.017. (H) Chromosomal interactions are inhibited at loci with methylated DNA. Interactions present in Col-0 were plotted if one side was found in either low or high DNA methylation 250bp non-centromeric bins defined in S1K–S1M Fig. These were plotted as a ratio to the expected (calculated from random bins—dashed line). Bars represent data from combined replicates while points represent individual replicates color-coded as in Figure 1B. Significance calculated by a t-test of replicates, p≤0.05 for meCG, meCHG, and meCHH.

We first sought to test the hypothesis that RdDM affects the expression of genes located in close proximity to direct RdDM targets. In order to identify RdDM regulated genes we performed RNA-seq in the *ago4* mutant with three biological replicates. We tested whether these genes are directly regulated by AGO4 targeting to the promoter by examining the promoters of differentially expressed genes for the presence of AGO4 as identified by ChIP-seq. Although AGO4 binding sites are generally enriched upstream of genes [8], transcriptional start sites (TSS) of genes with differential expression are generally distant from AGO4 binding to chromatin (S1A Fig). In fact, only ~5% of differentially expressed genes had an AGO4 binding site within 2.5 kb upstream of the TSS (Fig 1A). Furthermore, this small overlap was not a result of ChIP-seq peak calling stringency as a less stringent cutoff did not improve the overlapping region (Fig 1A, dashed circle). It should be noted that 2.5 kb is likely an overestimation of putative promoter length in *Arabidopsis*, but demonstrates the lack of overlap between *ago4* differentially expressed genes and AGO4 binding sites. The large portion of differentially expressed genes without proximal AGO4 binding suggests that only a limited subset of RdDM-affected genes is controlled by RdDM within their putative promoters.

The manner by which RdDM can control gene expression from long distances is unclear. One possibility is that RdDM may directly target a transcriptional regulator and thereby change the transcription of multiple genes as an indirect downstream consequence. We tested an alternative, but not mutually exclusive hypothesis that RdDM may control the activity of distant regions via 3D chromatin organization. This could take advantage of existing chromatin interactions or involve alterations of chromatin interactions to regulate gene expression. To investigate these possibilities we performed chromosome conformation capture experiments followed by high throughput sequencing (Hi-C) [31,32]. We performed Hi-C in three biological replicates using the *Dpn*II restriction endonuclease for fragmentation (Fig 1B), and long-distance chromatin contacts were identified by two parallel approaches. First, we used a simple approach to identify potential long distance chromatin interactions based on filtering by sequencing read count and the distance between mapped ends (see Materials and methods). We refer to this method as simple interaction calling which was applied to individual biological repeats (Fig 1B). Analysis of Hi-C often involves combining replicates in order to obtain interactions at greater depth [33–36]. We first tested the reproducibility of the Hi-C contact maps between replicates by examining the correlation in interaction intensities between individual 25 kb bins (S1B Fig). Since we saw high correlation between replicates, we decided to identify contacts from combined replicates as an alternate, deeper view of the data (Fig 1B, S1B and S1C Fig).

In order to obtain high confidence / stringent interactions, we also used a published interaction caller—Fit-Hi-C with ICE normalization (iterative correction and eigenvector decomposition) (S1C Fig) [37,38] which is stringent but not sensitive enough to work with the limited sequencing coverage available for individual biological repeats. We examined the distribution of interaction distances found by each of these methods and found that Fit-Hi-C calls at 1 kb resolution generally represent longer distances due to its use of distance normalization (S1D Fig) [38]. However, Fit-Hi-C inherently does not support inter-chromosomal interaction calling while simple calling maintains these interactions (S1E Fig). Using both methods we can evaluate results independent of the sensitivity / stringency of interaction calls. As the less stringent calling method allows comparison between biological replicates, these interaction calls are used throughout the manuscript and confirmation by Fit-Hi-C is provided in the supplemental data.

### Features of wild-type chromatin organization in *Arabidopsis thaliana*

We started our analysis with testing if our Hi-C datasets recapitulate known features of chromosome organization in *Arabidopsis*. One aspect of Hi-C is approximately exponential decay of interactions with increasing distance. We plotted this decay for previously published Col-0 (wild-type) Hi-C and the corresponding decrosslinked control (Fig 1C, blue and grey) [29]. When we assigned our contacts to *Dpn*II restriction fragments, our datasets showed much slower decay than the decrosslinked control (Fig 1C, orange) indicating that our Hi-C data may reflect real interactions.

Some studies have suggested the presence of topologically associating domains (TADs) in *Arabidopsis* [29], while others have not been able to confirm the presence of TADS [28,39]. Likewise, we cannot clearly see TADs in our data (S1F Fig). This could be partially due to the different developmental stages used in our study and in each of the other Arabidopsis Hi-C studies [28,29,39]. Additionally, in an effort to create Hi-C libraries with which we could compare to AGO4 ChIP-seq, we used low crosslinking conditions (0.5% formaldehyde) which may also explain any visual differences in broad raw Hi-C heatmaps (S1F Fig).

To confirm the quality and reproducibility of our Hi-C data, we tested whether we could recapitulate other known and reproducible features of wild-type *Arabidopsis* chromatin organization. A broad feature of *Arabidopsis* Hi-C is the self-association of chromosome arms [28,29,39]. To test this, we examined broad associations along chromosomes by calculating the correlation of interactions between each 1 Mb bin. We then performed a principle component analysis and took the first component (eigenvector) to calculate the clustering of genomic bins, a strategy which was originally employed in human cells [31]. Eigenvector analysis of the Hi-C data indicates two separate clusters corresponding to chromosome arms (Fig 1D, S1G Fig). This indicates that regions on chromosome arms preferentially associate with other regions on the same arm rather than between arms.

Another feature typically seen by Hi-C is an enrichment for inter-telomeric association [28,29,39]. Similar to others, when we examined telomeres we detected higher association of telomeres with each other than with the neighboring regions (Fig 1E). Additionally, strong inter-chromosomal interactions between centromeres (including pericentromeric regions) are characteristic of Hi-C data [28,39] which we also find in our data (Fig 1F). We conclude that our Hi-C data recapitulate several previously identified features of *Arabidopsis* chromatin organization and despite limited sequencing depth are a good measure of chromatin interactions.

### Chromatin interactions are inhibited at repressive chromatin

It is interesting that centromeres, which have high levels of repressive chromatin modifications, engage in strong chromatin interactions (albeit inter-chromosomally). This suggests that chromatin with repressive modifications could be more likely to engage in specific chromosomal interactions in *Arabidopsis*. To test whether repressed chromatin outside of centromeric or pericentromeric regions is also associated with long range chromosomal interactions, we first identified non-centromeric sites that display marks of active or inactive chromatin. Ratios of the levels of the active mark H3K4me2 to the inactive mark H3K9me2 [40,41] in equal sized bins display a bimodal distribution which we used to identify active and inactive chromatin (S1H Fig). We then counted the frequency of identified chromatin interactions associated with active or inactive chromatin. To compare replicates with different sequencing depths and to test for enrichment, we normalized by interactions occurring in randomly permutated non-centromeric bins (expected). From these data we find that non-centromeric inactive regions engage in long distance chromosomal interactions less often than active regions and less often than random bins (expected) (Fig 1G). This difference was consistent between the combined dataset (bar) and individual biological repeats (dots), which shows that it exceeds the biological variation. Similar analyses with Fit-Hi-C called interactions and with data from Wang et al displayed the same pattern (S1I Fig) [29]. We thought it was possible that the lower numbers of interactions in inactive chromatin could stem from inaccessibility to *Dpn*II due to crosslinked compacted chromatin. To test the efficiency of *Dpn*II at inactive regions, we examined a previously published decrosslinked control Hi-C library [29] and saw no difference in read distribution between active and inactive chromatin (S1J Fig). This indicates that *Dpn*II is likely to have the same activity despite the chromatin state and thus the digestion efficiency cannot fully explain the lower number of interactions anchored in inactive chromatin. Therefore, the data indicate that non-centromeric regions with repressive histone modifications are overall inhibited from participating in chromosomal interactions, which is consistent with data previously reported for *Arabidopsis* [28,29,39,42] and for other organisms [21,26,27].

To test the effects of DNA methylation on chromatin interactions we examined genomic bins (250 bp bins) with high or low levels of DNA methylation in the CG, CHG, and CHH sequence contexts (S1K–S1M Fig). We found that highly methylated bins participate in fewer total long-range chromatin contacts than bins with low levels of DNA methylation especially in the non-CG context (Fig 1H, S1N and S1O Fig). These differences were consistent between biological repeats. This observation is also consistent with previously published Hi-C in *Arabidopsis* [28,29]. This indicates that methylated regions, especially in the CHH context, are less likely to engage in detectable chromatin interactions.

### RdDM inhibits chromosomal interactions

**Fig 2:**
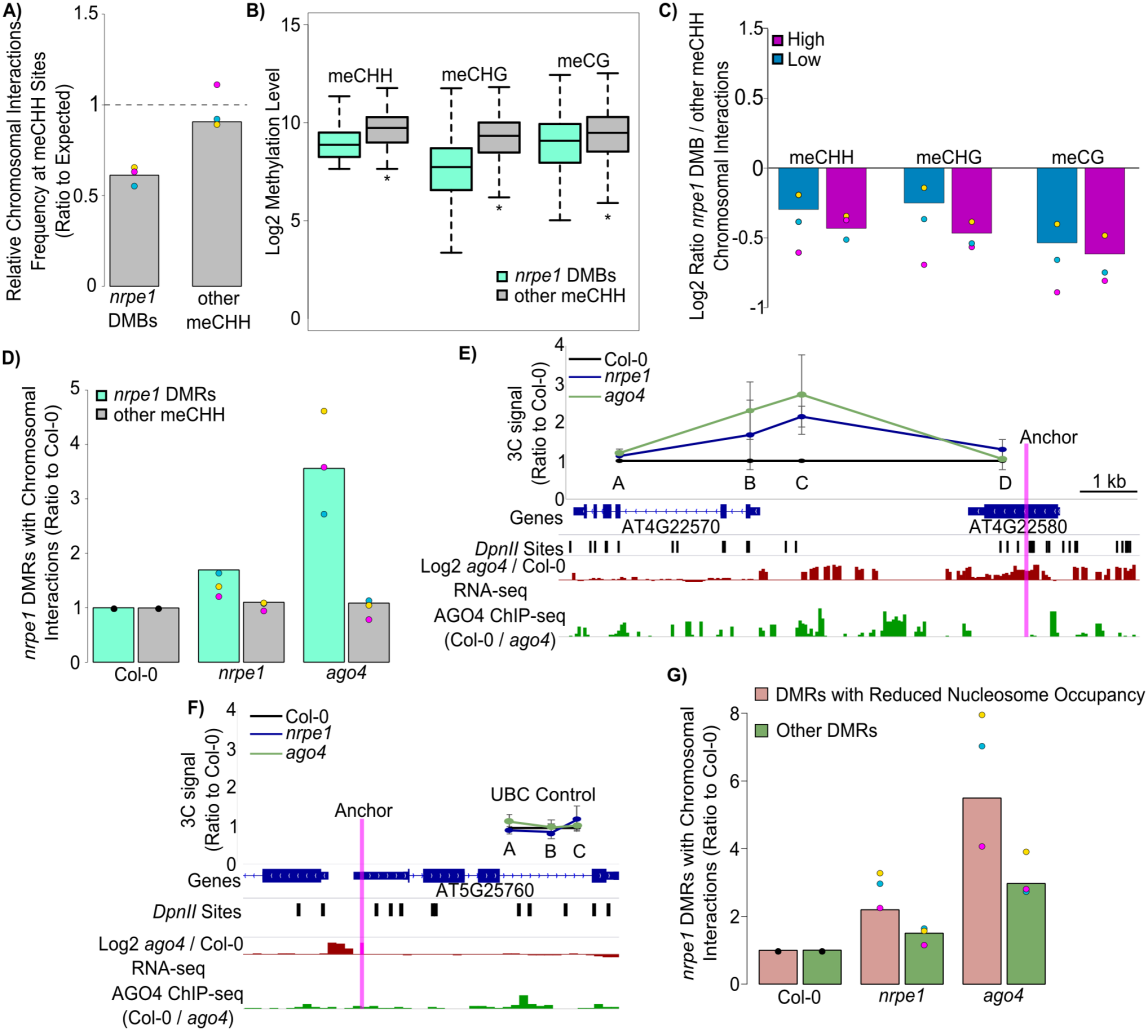
RdDM is associated with inhibited chromosomal interactions. (A) Chromosomal interactions are inhibited at sites with Pol V dependent DNA methylation. Interactions present in Col-0 were plotted if one side was found in sites with high CHH methylation as defined in S1M Fig categorized as those reduced in *nrpe1* (*nrpe1*/Col-0 < 0.25) (*nrpe1* DMBs) and those unchanged (*nrpe1*/Col-0 > 0.75) (other meCHH). These were plotted as a ratio to the expected (calculated from random bins). Bars represent data from combined replicates while points represent individual replicates color-coded as in Fig 1B. Significance calculated by a t-test of replicates, p≤0.05. (B) Methylation levels differ at RdDM sites. Methylation in each context for *nrpe1* DMBs (Differentially Methylated Bins) and other meCHH bins. * indicates p value ≤ 0.05 from a two-tailed t-test. (C) Interaction inhibition corresponds to RdDM. Comparison of *nrpe1* DMBs to other meCHH bins with matched methylation levels. Low (blue) indicates bins with methylation levels below the median *nrpe1* DMB level and is applied to both bin categories. High (purple) indicates loci with methylation levels above the median of other meCHH bins and is applied to both bin categories. Bars represent data from combined replicates while points represent individual replicates color-coded in Fig 1B. (D) Comparison of chromosomal interactions from RdDM target loci in Col-0, *nrpe1*, and *ago4*. Plot shows the numbers of *nrpe1* DMRs (teal) with detectable chromosomal interactions in *nrpe1* or *ago4* relative to Col-0. Also shown are CHH regions that do not overlap *nrpe1* DMRs (grey). Data for various interaction score cutoffs are shown in S2J Fig. Color coding is the same as in Fig 1B. Significance calculated by a t-test of replicates between DMRs and other meCHH sites, p≤.05 in *nrpe1* and in *ago4*. (E) 3C of a locus found by Hi-C. 3C was performed for an interacting site displaying an increase in looping in *nrpe1* (blue) and *ago4* (green) and analyzed by qPCR. A, B, and D indicate primer locations of fragments surrounding the interaction occurring between the anchor (pink) and C. Signals were normalized by an internal loading control amplifying a region without *Dpn*II sites. Error bars represent the standard error between 5 biological replicates. Gene annotations and *Dpn*II sites are shown below. AGO4 ChIP-seq signal in 50 bp bins (Col-0 / *ago4*) in green is shown. The log2 fold change in RNA-seq signal between *ago4* and Col-0 is also shown (red). (F) 3C of UBC control. 3C was performed for an anchor and three sites displaying no increased looping in *nrpe1* (blue) or *ago4* (green) and analyzed by qPCR. Signals were normalized by an internal loading control amplifying a region without *Dpn*II sites. Error bars represent the standard error between 5 biological replicates. Gene annotations, *Dpn*II sites, and primer locations are shown. AGO4 ChIP-seq signal in 50 bp bins (Col-0 / *ago4*) in green is shown. The log2 fold change in RNA-seq signal between *ago4* and Col-0 is also shown (red). (G) Nucleosomes enhance inhibition of chromosomal interactions. The number of *nrpe1* DMRs with detectable chromosomal interactions in *nrpe1* or *ago4* relative to Col-0 DMRs were categorized as overlapping a nucleosome reduced in *nrpe1* (MNase-seq Col-0/*nrpe1* > 2) (peach) or not (green). Color coding for individual replicates is the same as in Fig 1B.

The depletion of chromosomal interactions at genomic regions with repressive chromatin modifications (Fig 1GH) suggests that pathways involved in establishing heterochromatin may also repress chromatin interactions. To test whether the activity of RdDM is correlated with the inhibition of chromatin interactions we examined interactions at bins with high levels of CHH methylation in Col-0 wild-type, which is reduced in *nrpe1*, a mutant in the largest subunit of Pol V. These *nrpe1* differentially methylated bins (*nrpe1* DMBs) had fewer interactions than the randomized control (Fig 2A, S2A Fig). In comparison, bins where CHH methylation is unchanged in *nrpe1* did not show the same inhibition (Fig 2A, S2A Fig). These differences were consistent between biological repeats. This indicates that long range chromosomal interactions are inhibited at RdDM target loci.

Due to the differences in contact frequency between *nrpe1* DMBs and other meCHH bins, we checked whether features other than RdDM distinguish the two categories. Although methylation levels were high in both categories (due to their definition, see Materials and methods), differences between the two are evident such that unchanged meCHH bins generally have higher methylation than *nrpe1* DMBs (Fig 2B). We tested whether these differences in methylation levels between *nrpe1* DMBs and other meCHH bins were responsible for the differences in interaction signal. This was done by matching methylation levels between the categories. First, we compared *nrpe1* DMBs and other meCHH bins where both categories had methylation levels lower than the median *nrpe1* DMB level (Fig 2B). These matched *nrpe1* DMBs still had less chromatin interactions despite the similar methylation status (Fig 2C—blue). Next, we compared *nrpe1* DMBs to other meCHH bins where both had methylation levels higher than the median level in the other meCHH bins (Fig 2B). Again, we found that matched *nrpe1* DMBs had less chromatin interactions despite the similar methylation status (Fig 2C—purple). In each case the *nrpe1* DMB interaction signal was lower than other meCHH bins irrespective of methylation level or sequence context (Fig 2C). We performed the same analysis comparing H3K9me2 / H3K4me2 levels but found similar results (S2B and S2C Fig). Overall, this indicates that long range chromatin interactions are inhibited on loci targeted by RdDM and that this inhibition is probably not a result of chromatin signatures.

To further test the effects of RdDM on long range chromosomal interactions we performed Hi-C in *nrpe1* and *ago4* mutants, both of which are defective in RdDM [43–46]. We performed each Hi-C experiment in three replicates and found that the replicates correlated well with each other (S2D and S2E Fig). We examined Hi-C contact maps in these mutants and found that the overall organization of chromatin was similar to that of Col-0 (wild-type) (S2F Fig) and that *nrpe1* and *ago4* contacts displayed decay rates similar to Col-0 (S2G Fig). To test the effects of RdDM on chromatin interactions, instead of equal sized bins we focused our analysis on published *nrpe1* DMRs which are short CHH methylated regions where methylation is lost in *nrpe1* (S2H Fig) [14]. We examined high confidence chromosomal interactions detected in Col-0, *nrpe1* and *ago4* and observed increased interaction signals at *nrpe1* DMRs in *nrpe1* and *ago4* mutants compared to Col-0 wild-type, but no difference at other methylated regions (Fig 2D). This trend was also seen by Fit-Hi-C (S2I Fig). Furthermore, the increase in interactions in *nrpe1* and *ago4* was more pronounced when we consider interactions called at higher confidence (S2J Fig). These data indicate that RdDM represses the formation of chromosomal interactions.

We examined a locus displaying increased Hi-C interactions in *nrpe1* and *ago4* and tested the interaction by 3C-qPCR. This verified the mutant-specific increase in interaction between the loci identified by Hi-C (Fig 2E, Anchor—C) while primers on either side showed no enrichment (Fig 2E, primer A and D). This increased interaction occurred between a site enriched in AGO4 ChIP-seq signal (Fig 2E—green) and showed increased RNA-seq signal in *ago4* (Fig 2E—red). We performed this same analysis at a control locus which showed no increase in interactions by Hi-C and saw no increase by 3C (Fig 2F). Additionally, by Hi-C we found an increase in an extremely long distant interaction that occurs between two loci more than 8 Mb apart (Chr4: 654624–8811559). We tested whether this increase was also identifiable by 3C-qPCR and found that these loci interact more strongly in *nrpe1* and *ago4* (S2K Fig—box). We tested several other *Dpn*II fragments closer to the anchor and found no increase in interactions (S2K Fig) indicating that the long distance interaction is specific to that site. These results verify that increases in interactions occur in *nrpe1* and *ago4* at RdDM targets, and at least some increased interactions occur between AGO4 bound loci and genes differentially expressed in *ago4*.

Chromosomal interactions have been shown to be affected by nucleosome positioning [47,48] and nucleosome positioning is known to be an aspect of chromatin that is altered by RdDM [12]. We tested whether nucleosome positioning corresponds to changes in chromosomal interactions at *nrpe1* DMRs. DMRs with nucleosomes reduced in *nrpe1* show a greater increase in chromosomal interactions in *nrpe1* and *ago4* than those with unchanged nucleosomes (Fig 2G) although this difference was not present when examining the more stringent Fit-Hi-C chromatin interactions (S2L Fig). In any case, while nucleosome positioning may contribute, it cannot fully explain increased interactions in *nrpe1* and *ago4*, as increases in chromatin interactions are still present at DMRs where nucleosomes are unchanged in *nrpe1* (Fig 2G and S2L Fig). These data indicate that RdDM inhibits the formation of long range chromosomal interactions at its target loci by a mechanism independent of nucleosome remodeling.

### Gene activity corresponds to interactions with transcription factor binding sites

**Fig 3:**
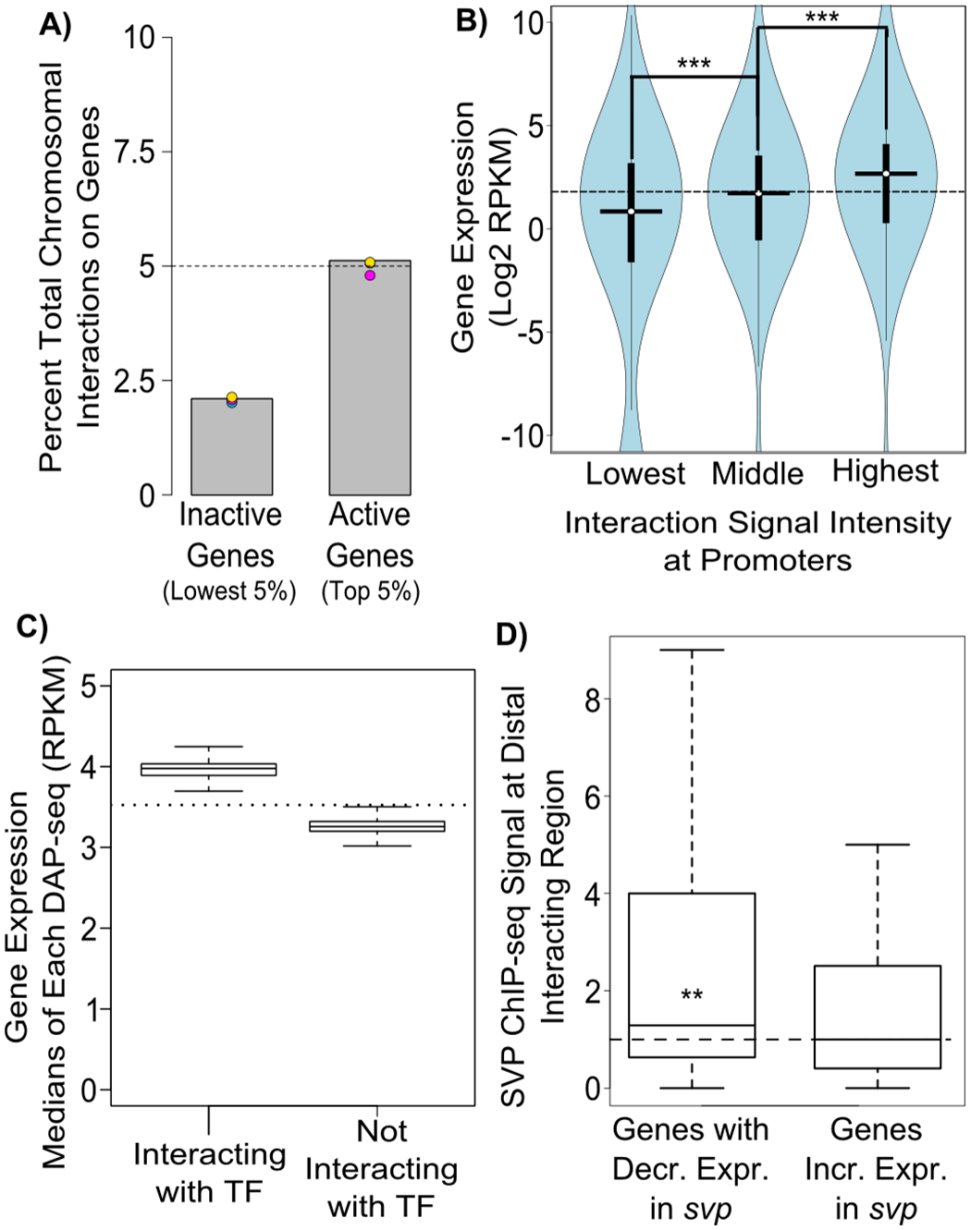
Gene activity corresponds to chromosomal interactions with transcription factor binding sites. (A) Inactive genes are less likely to engage in chromosomal interactions. The number of chromosomal interactions is plotted as a percentage of total interactions on genes. Inactive and active genes are the 5% of genes with the lowest or highest RNA-seq signals in Col-0 with promoters that are mappable in Hi-C (see Methods). Expected value from an even distribution of interactions on genes is indicated by a dashed line. Color coding is the same as in Fig 1B. Significance score calculated by a t-test of replicates, p≤0.01. (B) Promoter chromosomal interactions correspond to gene expression. Gene expression (calculated from RNA-seq) is plotted for genes with promoters at the lowest 5%, 5% centered at the middle, and the highest 5% for interaction signal in the combined dataset in Col-0. Horizontal and vertical bars indicate median and +/- 1 quartile respectively. Dashed line indicates average gene expression value for all genes. RPKM—reads per kilobase per million. *** p<0.001 Wilcoxon rank-sum test. (C) Genes connected to transcription factors have higher expression levels. RNA-seq based expression counts (RPKM) for genes connected to each transcription factor as detected by DAP-seq compared to the median expression of genes without interactions. The median values derived from each DAP-seq experiment are plotted. Dashed line indicates median expression of total genes. (D) Genes with reduced expression in *svp* interact with SVP binding sites. ChIP-seq signal of SVP (SVP-GFP / Col -0 RPM) at 250bp regions which loop to genes decreased or increased in *svp* mutant. Dashed line indicates no ChIP-seq enrichment. ** p<0.01 Wilcoxon rank-sum test.

Our observation that RdDM inhibits the formation of long range chromosomal interactions suggests that chromosome organization may be a general mechanism used by RNA-mediated transcriptional silencing pathways to control gene activity. However, it is unknown to what extent chromosome organization contributes to gene regulation in *Arabidopsis*. To test if the presence of long range chromosomal interactions may be functionally relevant to gene expression, we first checked whether there is a relationship between gene expression levels and the presence of chromosomal interactions. We used RNA-seq in Col-0 wild-type to identify genes that are either inactive or highly expressed and examined how often their putative promoters (defined as 1kb upstream of TSS) are engaged in chromosomal interactions. Inactive genes engage in interactions less often than active genes, and less than expected from random genes (Fig 3A, S3 Fig). This difference was consistent between biological repeats. We further tested the correlation between chromatin interactions and gene expression by analyzing genes with varying levels of interaction signal intensity in their putative promoters. Genes with low numbers of interactions at their promoter regions had lower median expression levels and, conversely, genes with high numbers of interactions had higher median expression levels (Fig 3B). This indicates a positive relationship between gene expression levels and overall chromosomal interaction frequency in *Arabidopsis* which is similar to findings from other organisms [49–51].

It has been proposed that chromosomal interactions may influence gene expression by bringing distantly bound transcription factors into close proximity with promoters of target genes in other organisms [22,27,52]. It is, however, unknown if a similar mechanism exists in *Arabidopsis*. To test this possibility, we looked for evidence of interactions between transcription factor binding sites and genes. First we examined a large set of transcription factors and their putative binding sites identified by published DAP-seq data [53]. For each transcription factor in the DAP-seq dataset, we calculated the median expression value for genes with identified interactions that connected the gene to a transcription factor’s binding site. We also performed this analysis for genes not interacting with the transcription factor’s binding sites. We found that for each transcription factor, the median expression of interacting genes was higher than genes not interacting with the transcription factor. It was also higher than the genome-wide average (Fig 3C), which suggests that these interactions may be involved in controlling gene expression.

To find further evidence of long-range control by transcription factors, we then examined published ChIP-seq and gene expression data for a well-studied specific transcription factor, SVP [54]. We identified genomic regions which interact with genes that change expression in the *svp* mutant. SVP binding to chromatin detected by ChIP-seq was enriched at distant interacting sites. This enrichment was only seen on sites which interact with genes with decreased expression in the *svp* mutant (Fig 3D).

Overall, these findings indicate that higher gene expression levels in *Arabidopsis* are associated with the presence of long range chromosomal interactions. Although causality remains to be proven, this indicates that the principle of regulatory regions affecting distant genes by long range chromosomal interactions may be applicable to plant genomes.

### Gene activity is associated with inhibition of chromosomal interactions by RdDM

**Fig 4:**
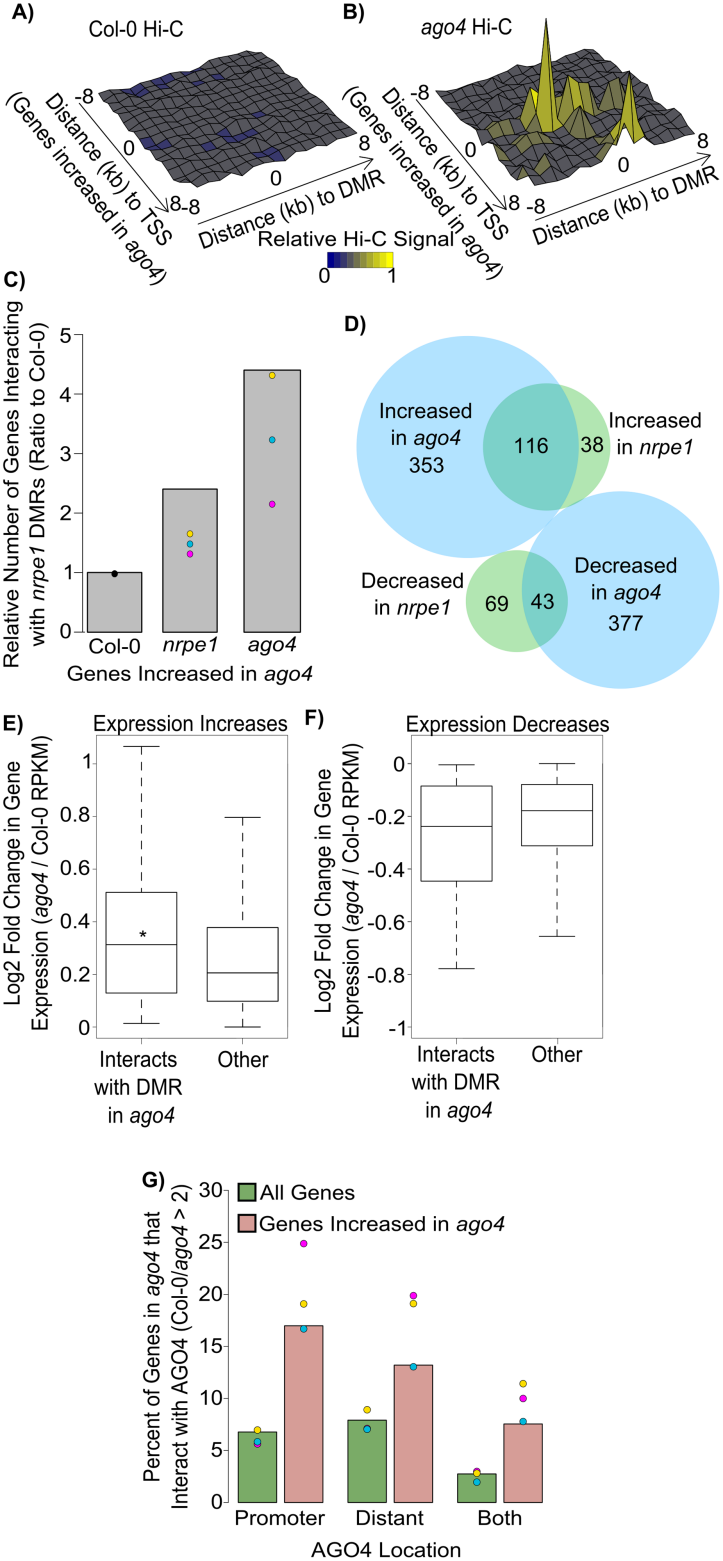
Repression of chromosomal interactions with RdDM targets corresponds to gene repression. (AB) Genes repressed by AGO4 are inhibited from interacting with RdDM targets. Surface plots of mean interaction signal in Col-0 (A) and *ago4* (B) for interactions between 1) regions surrounding TSS’s of genes with expression increased in *ago4* (y-axis) and 2) regions surrounding *nrpe1* DMRs (x-axis). Height corresponds to average interaction scores (z-axis—see Methods). (C) Numbers of genes, with increased expression in *ago4*, which form chromosomal interactions to *nrpe1* DMRs. Interactions are plotted in *nrpe1* or *ago4* as a ratio to Col-0 for genes with increased expression in *ago4* and for random genes. Color coding is the same as in Fig 1B. (D) Expression changes are greater in *ago4* than in *nrpe1*. Venn diagram of differential gene expression calls in *ago4* (blue) and *nrpe1* (green). (E) Increased interaction with *nrpe1* DMRs corresponds to increased gene expression. The positive log2 fold change in gene expression (RNA-seq *ago4* / Col-0) is plotted for genes which do not show increased interactions to *nrpe1* DMRs in the *ago4* mutant and for genes which do show increased interactions to *nrpe1* DMRs in the *ago4* mutant. The combined interaction dataset was used. RPKM—reads per kilobase per million. * p<0.05 Wilcoxon rank-sum test. (F) Increased interactions with *nrpe1* DMRs corresponds to decreased gene expression. The negative log2 fold change in gene expression (RNA-seq *ago4* / Col-0) is plotted for genes which do not show increased interaction to *nrpe1* DMRs in the *ago4* mutant and for genes which do show increased interactions to *nrpe1* DMRs in the *ago4* mutant. The combined interaction dataset was used. (G) AGO4 may bind both gene promoters and distant regulatory regions. Plot shows genes with chromosomal interactions in *ago4*, which have AGO4 binding to the promoter (1kb upstream of transcriptional start sites), to the distant regulatory regions (detected by Hi-C), or to both. Genes increased in *ago4* (peach) are compared to total genes (green). Color coding for individual replicates is the same as in Fig 1B.

Our observations that RdDM inhibits long range chromosomal interactions and that long range interactions exist between genes and TF binding sites suggest that genes affected by RdDM may preferentially interact with distant genomic regions targeted by RdDM. To test this possibility, we analyzed contact map profiles around TSS’s of genes that are repressed by RdDM (increased expression in *ago4*). We tested if there was enrichment for long distance connections (> 10kb) to RdDM sites (*nrpe1* DMRs). Average contact signal in Col-0 is not enriched between RdDM affected genes and *nrpe1* DMRs (Fig 4A) consistent with repression of contacts at *nrpe1* DMRs in Col-0 (wild-type) (Fig 2). We next performed the same analysis in the *ago4* Hi-C sample and observed an enrichment of contacts between differentially expressed genes and DMRs (Fig 4B). These interactions occur within 1–2 kb of the TSS’s on one end (y-axis) and are centered around *nrpe1* DMRs on the other end (x-axis). This indicates that RdDM inhibits long-range chromosomal interactions between promoters of affected genes and RdDM sites similar to what we saw by 3C (Fig 2E). Because of the narrow range around the TSS, we conclude that this increase in interaction is probably not simply a result of a general change in chromatin organization, but is more likely caused by specific chromatin interactions between RdDM sites and promoters (Fig 4B). To further examine the effect of RdDM on promoter specific interactions, we examined the relative number of genes that are repressed by RdDM and are connected to *nrpe1* DMRs in each genotype. Chromosomal interactions detected in *nrpe1* and *ago4* identified larger numbers of genes connected to *nrpe1* DMRs than in Col-0 wild-type (Fig 4C). This increase was visible in all three biological repeats (dots), which shows that the observed effects are greater than the biological variation, although it should be noted that this is supported by a relatively small number of genes (S2 Table). However, no enrichment was observed when we analyzed random sets of genes (S4A Fig). It is noteworthy that this effect is greater in the pooled data (Fig 4C—grey bars) than in each of the replicates (Fig 4C—points) suggesting that the sequencing depth is a limiting factor in detecting these interactions. These data indicate that a subset of genes affected by RdDM preferentially interacts with distant genomic regions targeted by RdDM and that these interactions are repressed by RdDM in Col-0 wild-type.

Interestingly, *ago4* had a stronger effect on long range chromosomal interactions than *nrpe1* (Fig 4C, see also Fig 2D). We compared the effects of *nrpe1* and *ago4* mutation on gene expression and found a stronger effect in *ago4* (Fig 4D, S4B Fig), which is consistent with the larger effect to chromatin interactions (Fig 4C). Furthermore, this indicates that although both Pol V and AGO4 are required for RdDM, *nrpe1* and *ago4* mutants may differentially affect chromatin and gene expression.

The observation that genes with increased expression in *ago4* also gain interactions with RdDM targets (DMRs) suggests that RdDM may control gene expression by changing chromatin organization. To further test this model, we analyzed all protein coding genes and categorized them based on the presence or absence of chromosomal interactions (detectable in *ago4*) to direct RdDM targets (*nrpe1* DMRs). We then separated out genes that show any increases in expression and genes that show any decreases in expression. Genes that interact with RdDM targets in *ago4* were more likely to have increased expression in the *ago4* mutant compared to genes which do not interact with RdDM targets (Fig 4E). Similarly, reductions in gene expression in *ago4* were also more frequent among interacting genes, but this trend was not statistically significant (Fig 4F). Overall the effects on genes activated by RdDM (expression reduced in *ago4*) were lower than on genes repressed by RdDM (expression increased in *ago4*) (S4A Fig, Fig 4C). These observations further confirm the association between the effects of RdDM on long range chromosomal interaction and gene expression levels.

Changes in gene expression observed in the *ago4* mutant may be explained by two non-mutually exclusive mechanisms (in addition to possible indirect effects). First, RdDM directly working on gene promoters or promoter proximal elements may affect transcription locally without the involvement of chromosomal interactions. Second, RdDM targets on distant regulatory regions may affect gene expression via chromosomal interactions. To distinguish between those possibilities, we tested if AGO4 preferentially localizes to both distant and promoter ends of inhibited interactions. For these purposes, we define distal regulatory regions as located at least three *Dpn*II restriction sites away from the gene or its proximal regulatory region (1kb upstream of TSS) and connected by detectable chromosomal interactions from Hi-C in the *ago4* mutant. Many regulated genes with inhibited interactions only had AGO4 on either the promoter or distal region suggesting that either is sufficient for gene regulation (Fig 4G). We also observed no prominent distance or orientation preference between genes and RdDM targets (S4C Fig). Although causality between RdDM-repressed looping and gene expression levels remains to be shown, this is consistent with RdDM affecting gene expression by a combination of local and long range effects.

Together, these results show that RdDM negatively affects long range chromosomal interactions between RdDM-repressed genes and distant direct RdDM targets. This could be explained by RdDM-mediated changes in gene expression affecting chromosomal interactions or RdDM affecting both gene expression and chromosomal interactions in parallel. We propose that RdDM contributes to gene repression by inhibiting interactions between genes and distant regulatory regions.

## Discussion

**Fig 5:**
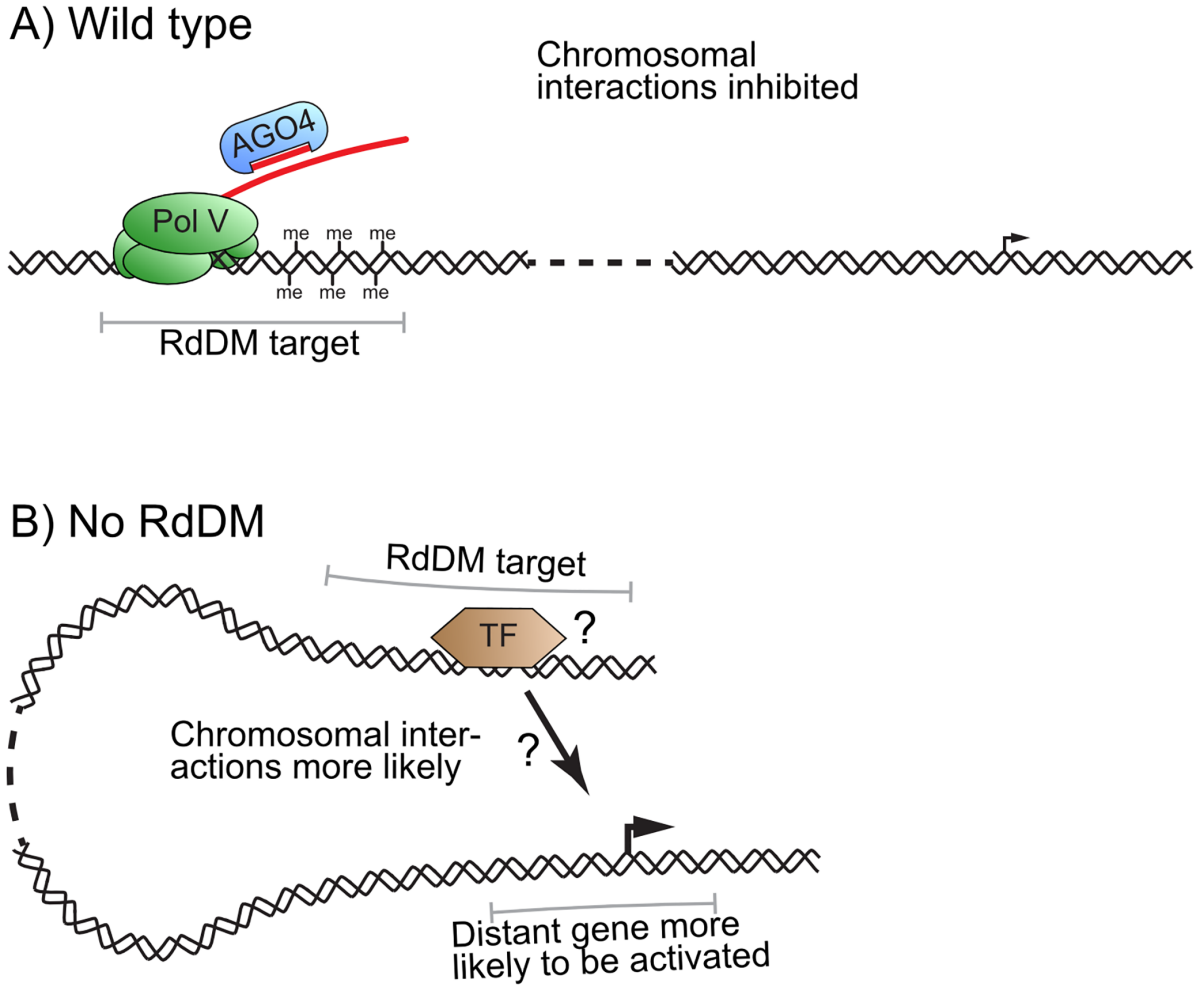
Speculative model of gene regulation by RdDM. (A) In wild-type plants Pol V and AGO4 bind to chromatin and help establish repressive chromatin marks at regulatory regions. These chromatin modifications inhibit chromosomal interactions between genes and distant regulatory regions. (B) When RdDM is not functional, chromosomal interactions are able to occur between promoters and distant regulatory regions bound by transcription factors (orange), thereby affecting gene expression.

We propose a model, where RdDM represses long range chromosomal interactions. In wild-type Col-0 plants, regions directly targeted by RdDM have high levels of DNA methylation, repressive histone modifications, and stabilized nucleosomes. At these sites, RdDM inhibits the formation of chromosomal interactions (Fig 5—wild-type). When RdDM is not active in the *ago4* mutant or under specific environmental conditions, repressive chromatin marks / RdDM components are not present and chromosome interactions are more likely to occur (Fig 5—no RdDM). We further speculate that in some cases these interactions may bring distant enhancers with associated transcription factors to the proximity of gene promoters and affect gene expression.

Looping between specific chromosomal regions is a conserved process found by chromosome conformation capture from bacteria to mammals [50,51,55–57] and is believed to contribute to the regulation of gene expression [22,58,59]. In *Drosophila*, where enhancers have been identified genome-wide, gene expression correlates well with promoter—enhancer contacts [60]. In human cells, Capture Hi-C also found that promoters of active genes participate in chromatin interactions more than inactive genes [61]. Similarly, yet independently, it was found that sites with higher RNA Pol II signal are more likely to participate in chromatin interactions [62]. Our data are consistent with this mechanism existing also in plants, in that high levels of gene expression correspond to higher confidence chromatin interactions (Fig 3A and 3B) and that differential gene expression correlates with differential chromatin contacts (Fig 4A–4C).

Our model where RdDM inhibits chromatin interactions leads to the important question of which features of RdDM directly affect looping. This may be unknown or untested chromatin modifications; however, DNA methylation, H3K9me2 / H3K4me2, and nucleosome position by themselves cannot explain differences in chromatin interactions (Fig 2C and 2G and S2C Fig). It is more likely that chromosomal interactions are directly mediated by specific transcription factors or architectural proteins that are unable to bind in the presence of RdDM. Alternatively, transcription may be an important part of chromatin organization, and differential expression could directly alter chromatin interactions.

An interesting aspect of our gene expression analysis is the difference between *nrpe1* and *ago4* (Fig 4D, S4B Fig). This is especially well supported by simple interaction calling, where *ago4* has a more prominent effect at RdDM sites than *nrpe1* (Fig 2D and 2G). One possibility is that *nrpe1* and *ago4* affect chromatin modifications differently at some loci. This is consistent with AGO4 binding RNAs produced by RNA polymerases other than Pol V but not engaging in RdDM [63]. A more attractive explanation is that the act of Pol V transcription in the absence of AGO4 has an effect independent of repressive chromatin modifications. In this scenario Pol V transcripts may act to promote long distance interactions as has been suggested for non-coding RNA in other organisms [52,64]. Supporting this model, several proteins required for chromatin interactions such as Mediator, Cohesin, and CTCF have been shown to interact with RNA [65–67] and it is tempting to speculate that Pol V-produced lncRNA acts in a similar manner.

It is important to note that our analysis shows genome-wide trends. Therefore, substantial locus-specific variation is still expected to occur. For example, a recent report demonstrated that RdDM and other chromatin mechanisms may actually facilitate the formation of a specific chromosome loop between the methylated *APOLO* locus and the *PID* gene in response to a plant hormone auxin [68]. However, it should be noted that while this locus is methylated in wild-type (Col-0), the methylation status at the *APOLO* loop anchor is unchanged in RdDM mutants (*nrpe1* and *ago4*) (S5 Fig). Furthermore, H3K9ac does not increase in the RdDM mutant at the APOLO loop anchor [68]. This suggests the presence of unknown contributors or compensators for the regulation of this locus aside from Pol V and AGO4. Additionally, the loop between *APOLO* and *PID* occurs at a distance of 3.5 kb apart, which is difficult to detect by Hi-C. Our study mainly focused on longer-range chromosomal interactions and may thereby miss shorter contacts. Thus, it is possible that RdDM may both inhibit and stabilize chromosomal interactions depending on their distance and/or other variables.

It should also be noted that chromosome conformation capture (Hi-C), relies on *in vivo* crosslinking and ligation, which are not fully understood and should be carefully interpreted. We attempted to mitigate some of the limitations of Hi-C by comparing biological repeats and pooled raw data, and by utilizing two different methods of interaction calling. We also performed comparative analysis with ChIP-seq, which involved a similar crosslinking step. Additionally, our findings do not rely only on wild-type conditions, but also include comparing different genetic backgrounds.

The results presented here indicate that RNA-mediated transcriptional silencing can affect several aspects of chromatin structure including not only chromatin modifications and nucleosome positioning but also long-range chromosomal interactions. This further demonstrates that RNA is a master regulator of chromatin structure.

## Materials and methods

### Plant material

*Arabidopsis thaliana* mutant lines *nrpe1 (nrpd1b-11)* and *ago4 (ago4-1* introgressed into Col-0) were described previously [45,69,70]. Seedling tissue was used in all experiments and only datasets from the same tissue type were used in analysis.

### 3C and Hi-C sample preparation

*Arabidopsis* seedlings were grown for 2 weeks under long day conditions after which above ground tissue was harvested and cross-linked in 0.5% formaldehyde as previously described [71]. Nuclei were extracted using the same protocol as ChIP [71], followed by *Dpn*II digestion, ligation, and purification of chromatin contacts similar to [72]. Following this protocol, nuclei were washed in 1.2x *Dpn*II buffer, resuspended in 550 μl 1.2x *Dpn*II buffer with 0.26% SDS, and then incubated for 20 min at 65°C followed by 20 min at 37°C with gentle shaking. Triton-X 100 was added to 1.6% with incubation for 60 min at 37°C. Chromatin was digested with 750 units *Dpn*II overnight at 37°C. After digestion, samples were incubated in 1% SDS for 20 min at 65°C. Samples were then diluted in 10 ml 1x T4 DNA ligase buffer (NEB) supplemented with 0.75% Triton-X 100 and incubated at 37°C for 60 min. Ligation was performed with 600 units T4 DNA ligase for 5 hours at room temperature. Decrosslinking occurred with 600 μg Proteinase K at 65°C overnight, followed by addition of 300 μg RNase A for 30 min at 37°C. DNA was then isolated using phenol:chloroform. Libraries were prepared and sequenced by the University of Michigan Sequencing Core. Primers used in 3C can be found in S1 Table.

### Hi-C data analysis

Each end of paired-end reads with unique alignment was mapped to the *Arabidopsis* genome (TAIR10) using Bowtie and then paired. Reads were assigned to 1 kb bins and significant intra-chromosomal interactions more than 3 kb apart were called by Fit-Hi-C with ICE (Fig 1B) [37,38]. These Fit-Hi-C calls were further filtered by estimating a false discovery rate (FDR). This FDR was calculated by permutating p-values such that in a random set of interactions with equal size only .05 were present at or below the p-value cutoff. In essence all called peaks had stringent enough p-values such that they would not be likely to appear in random sets. Alternatively, to obtain a looser view of interactions, the genome was divided into 250bp windows (bin-mapping) and interactions between windows were counted keeping only those with more than three *Dpn*II sites apart (Fig 1B). This was done not only to account for non-uniform cleavage distances, but to allow overlap of uniform datasets such as H3K4me2/H3K9me2 bins or DNA methylation bins. The top 5% interactions (read counts) of the shortest bin distance, 4 bins apart, were kept and that maximum number was applied to all subsequent bins. This approach allowed keeping only the highest confidence short range interactions while maintaining long-range interaction events with a minimum of two reads supporting independent ligation events (S1C and S1D Fig). Interaction scores were calculated from the number of reads supporting the interaction between two 250bp bins multiplied by the ratio between the total number of reads to the total number of loops called in that sample. Pearson Correlation between replicates was done in 25 kb bins using contact counts between bins normalized by sequencing depth. Eigenvector was calculated at 1 Mb resolution using juicebox [34]. Telomere association was plotted using scores from inter-chromosomal interactions. Highly scoring interactions (value > = 10) were used for overlaps between features to compare between replicates unless otherwise indicated.

Overlaps with interaction counts were normalized to total interactions for each where applicable and we have provided the raw overlap counts and normalization scheme for each in S2 Table. Significance scores for each were calculated by a two-sided paired t-test among biological replicates. Genomic regions tested for overlap with Hi-C data were first filtered based on their mappability in Hi-C. This was done by keeping only those features with reads in the decrosslinked control from Wang et al.[29]. Filtered inactive and active regions were further checked to ensure similar digestion efficiency by comparing the read counts in the decrosslinked control (S1J Fig) [29]. This was to ensure that lower mappability was not affecting the results and to ensure that restriction digestion to get mapped reads in Hi-C was able to penetrate heterochromatic regions.

Interaction enrichment plots between promoters and DMRs (Fig 4A and 4B) were plotted by 1) Filtering out interactions <10 kb apart; 2) Keeping reads that overlap bins surrounding the TSS on one side and DMRs on the other; 3) Taking the average or sum of each interaction bin in the matrix of bins according to the distance from TSS and from DMR; 4) Repeating steps 1–3 with TSS’s connected to bins surrounding random regions to calculate a randomized average and subtracting these average values from the matrix. Z-axis values and color scores are then plotted from these values obtained, as a relative score from the minimum and maximum. Interaction overlaps between promoters of differential genes and distal *nrpe1* DMRs was taken using an interaction score > = 5 to obtain as many overlapping high quality interactions as possible.

### RNA-seq

RNA from *ago4* seedlings was isolated and rRNA-depleted in three biological repeats as described [12] and libraries were prepared by the University of Michigan Sequencing Core. Reads were mapped to the TAIR10 genome assembly using Tophat [73] and differential expression was called using EdgeR [74]. Previously published RNA-seq datasets from seedlings (Col-0 wild-type and the *nrpe1* mutant) [12] (GSE38464) were obtained from plants grown, harvested, isolated, rRNA-depleted, and sequenced in parallel to the *ago4* dataset. Overlaps in differential expression were calculated from EdgeR and plotted as a weighted Venn diagram using the Venneuler package in R.

### Overlap with transcription factors

DAP-seq transcription factor binding sites came from the GEO accession GSE60141 [53]. Fig 3C was generated by taking interactions overlapping promoters (1kb upstream of TSS) on one end and DAP-seq transcription factor peaks on the other or no DAP-seq peak for each transcription factor tested. The median RPKM values of genes interacting in each transcription factor DAP-seq reaction were then plotted as a boxplot and provided in S3 Table. The genome-wide median was produced from total genes.

SVP ChIP-seq data were downloaded from GEO accession GSE33120 [54]. Reads were then mapped to the TAIR10 genome and immunoprecipitation vs. control were counted in 250bp windows for comparison to Hi-C which was taken at a more stringent cutoff score of 15 to compensate for variations in ChIP-seq quality. TF binding sites were defined as at least four fold enrichment in sample vs. control. EdgeR was used to call differential expression for *svp* from GEO accession GSE32397 [54].

### Other datasets used in this study

Datasets for AGO4 ChIP-seq came from GSE35381 [8], MNase-seq came from GSE38401 [12] and ChIP-seq for histone modifications came from GSE37644 and GSE49090 [40,41]. DNA methylation data came from GSE39901 [13] and *nrpe1* DMR calls came from [14].

## Supporting information

S1 Fig Features of the Hi-C datasets. (A) AGO4 binds distant from differential genes. Histogram of distances (Log2) between TSS’s of differentially expressed genes and the closest upstream AGO4 binding site. Vertical lines indicate 1 kb and 2.5 kb putative promoter distance cutoffs. (B) Hi-C replicates correlate well. Pearson correlation (r) of individual Hi-C replicates. Plotted are contact counts between 25 kb bins normalized by the total contacts in each replicate. (C) Number of called significant interactions by Fit-Hi-C and a simpler (looser) calling method (see Methods). (D) Distances of called interactions. Histogram of significant interaction distances called by each method. (E) Inter-chromosomal interactions. Fraction of inter-chromosomal identified interactions in each genotype. (F) Contact plot of chromosomal interactions. Contact scores in 100 kb bins were calculated and plotted. Yellow to red indicates weak to strong interaction signal, blue indicates very strong interaction signal. Approximate centromere positions are noted by black squares. (G) Self-association of chromosome arms. Eigenvector plot of contact correlation along each chromosome. (H) Identification of active and inactive chromatin regions. H3K4me2 ChIP-seq signal [41] and H3K9me2 ChIP-seq signal [40] are plotted relative to each other in 250bp bins. RPM—reads per million. (I) Non-centromeric inactive chromatin is inhibited from forming chromosomal interactions. Alternate interaction calls (Fit-Hi-C) present in Col-0 from this study and interactions from Wang et al. (26) were plotted if one side was found in either inactive (blue) or active (peach) non-centromeric chromatin bins defined in Fig 1C. These were plotted as a ratio to the expected (calculated from random bins—black line). (J) Non-centromeric inactive and active chromatin have equal potential Hi-C efficiency. Boxplots represent the number of reads present in the reverse-crosslinked control from Wang et al. (26) overlapping identified inactive and active regions defined in Fig 1C. RPM—reads per million. (K) Distinct sites of CG methylation. CG methylation within 250bp bins is plotted. Dashed line indicates division used to define low and high methylation groups used in Fig 1H. (L) Distinct sites of CHG methylation. CHG methylation within 250bp bins is plotted. Dashed line indicates division used to define low and high methylation groups used in Fig 1H. (M) Distinct sites of CHH methylation. CHH methylation within 250bp bins is plotted. Dashed line indicates division used to define low and high methylation groups used in Fig 1H. (N) Chromosomal interactions are inhibited at sites with high DNA methylation. Fit-Hi-C interactions (Figure S1C, see Methods) present in Col-0 were plotted if one side was found in either low (blue) or high (red) DNA methylation 250bp non-centromeric bins defined in Figure S1K-M. These were plotted as a ratio to the expected (calculated from permutated random bins—dashed line). (O) Chromosomal interactions are inhibited at sites with high DNA methylation. Interactions present in Col-0 from Wang et al. (26) were plotted if one side was found in either low (blue) or high (red) DNA methylation 250bp non-centromeric bins defined in Figure S1K-M. These were plotted as a ratio to the expected (calculated from permutated random bins—dashed line).
(TIF)

S2 Fig RdDM inhibits chromosomal interactions at RdDM sites. (A) Chromosomal interactions are inhibited at sites with Pol V dependent DNA methylation. Fit-Hi-C called interactions present in Col-0 were plotted if one side was found in sites with high CHH methylation as defined in S1M Fig categorized as those reduced in *nrpe1* (*nrpe1*/Col-0 < 0.25) and those unchanged (*nrpe1*/Col-0 > 0.75). These were plotted as a ratio to the expected (calculated from random bins—dashed line). (B) Histone modifications at RdDM sites. Log2 ratio of H3K9me2 / H3K4me2 for nrpe1 DMBs (Differentially Methylated Bins) and other meCHH bins. (C) Interaction inhibition corresponds to RdDM. Comparison of *nrpe1* DMBs to other meCHH bins with matched H3K9me2/H3K4me2 levels. Low (blue) indicates modification levels below the median *nrpe1* DMB level and is applied to both bin categories. High (purple) indicates methylation levels above the median of other meCHH bins and is applied to both bin categories. Bars represent data from combined replicates while points represent individual replicates color-coded in Fig 1B. (D) Hi-C replicates in *nrpe1* correlate well. Pearson correlation (r) of individual Hi-C replicates. Plotted are contact counts between 25 kb bins normalized by the total contacts in each replicate. (E) Hi-C replicates in *ago4* correlate well. Pearson correlation (r) of individual Hi-C replicates. Plotted are contact counts between 25 kb bins normalized by the total contacts in each replicate. (F) Contact plot of *nrpe1* and *ago4* chromosomal interactions. Contact scores in 100 kb bins were calculated and plotted for *nrpe1* (top) and *ago4* (bottom). Yellow to red indicates weak to strong interaction signal, blue indicates very strong interaction signal. Approximate centromere positions are noted by black squares. (G) Decay curves of Col-0 Hi-C compared to *nrpe1* and *ago4*. Reads were aligned to *Dpn*II fragments and kept if >3 *Dpn*II sites apart. These were assigned to 250bp genomic bins and plotted. Col-0 (orange) compared to *nrpe1* Hi-C (blue) and *ago4* Hi-C (green). (H) Sizes of previously called *nrpe1* DMRs. Histogram depicting size distribution of *nrpe1* DMRs. (I) Comparison of chromosomal interactions from RdDM target loci in Col-0, *nrpe1*, and *ago4*. Plot shows the percentage of *nrpe1* DMRs with detectable Fit-Hi-C chromosomal interactions in Col-0, *nrpe1*, and *ago4*. (J) Comparison of looping from RdDM target loci in Col-0, *nrpe1* and *ago4*. The number of *nrpe1* DMRs with detectable chromosome looping in *nrpe1* (blue), or *ago4* (green) relative to Col-0 at different loop score cutoffs is plotted. Line represents combined data while points are individual replicates color-coded as in Fig 1B. (K) 3C of a long-range interaction. 3C was performed for an anchor (Chr4: 653624) and a site 8 Mb away (Chr4: 8811559) displaying increased looping in *nrpe1* (blue) or *ago4* (green) and analyzed by qPCR. Primers for intervening sites were also amplified as controls. Signals were normalized by an internal loading control amplifying a region without *Dpn*II sites. Error bars represent the standard error between 5 biological replicates. (L) Nucleosomes slightly enhance inhibition of chromosomal interactions. The number of *nrpe1* DMRs with detectable chromosomal interactions called by Fit-Hi-C in *nrpe1* or *ago4* relative to Col-0 DMRs were categorized as overlapping a nucleosome reduced in *nrpe1* (MNase-seq Col-0/*nrpe1* > 2) (peach) or not (green).
(TIF)

S3 Fig Gene activity corresponds to chromosomal interactions. Inactive genes are less likely to engage in chromosomal interactions. The number of chromosomal interactions is plotted as a percentage of total interactions on genes. Inactive and active genes are the 5% of genes with the lowest or highest RNA-seq signals in Col-0 with promoters that are mappable in Hi-C (see Methods). Expected value from an even distribution of interactions on genes is indicated by a dashed line.
(TIF)

S4 Fig Repression of genes corresponds to RdDM repressed looping. (A) Genes activated by AGO4 are slightly inhibited from interacting with *nrpe1* DMRs. Plot shows numbers of genes, with decreased expression in *ago4*, which form chromosomal interactions to *nrpe1* DMRs. Interactions are plotted in *nrpe1* or *ago4* as a ratio to Col-0 for genes with increased expression in *ago4* and for random genes. Color coding is the same as in Fig 1B. (B) *ago4* causes more drastic changes to gene expression than *nrpe1*. Log2 fold changes in *ago4* or *nrpe1* vs Col-0 from three biological repeats as calculated by EdgeR are plotted for called differential genes (blue) and total genes (grey). Diagonal line indicates a slope of 1, vertical and horizontal lines indicate no change for *nrpe1* or *ago4* respectively. (C) Loops between genes and DMRs are independent of distance and directionality. Chromosome loops established in *ago4* are plotted if one end lies within an *nrpe1* DMR and the other is in a gene promoter. Distance between ends and directionality from the transcriptional start site (TSS) is shown.
(TIF)

S5 Fig Locus specific variation in RdDM activity. View of the published *APOLO* loop showing DNA methylation in Col-0, *nrpe1*, and *ago4* [13]. meCG (yellow), meCHG (blue), and meCHH (orange) are shown. ChIP-seq signal enrichment for Pol V is also shown (green) [75].
(TIF)

S1 Table Oligonucleotides used for 3C-qPCR. (XLSX)

S2 Table Raw data used to generate figures shown in this study. (XLSX)

S3 Table Expression levels of genes looping to transcription factors. (XLSX)
