# Supplementary figures and images for: Long-range control of gene expression via RNA-directed DNA methylation
Source: PLoS Genet. 2017 May 5;13(5):e1006749. doi: 10.1371/journal.pgen.1006749 (PMC5438180; doi:10.1371/journal.pgen.1006749)

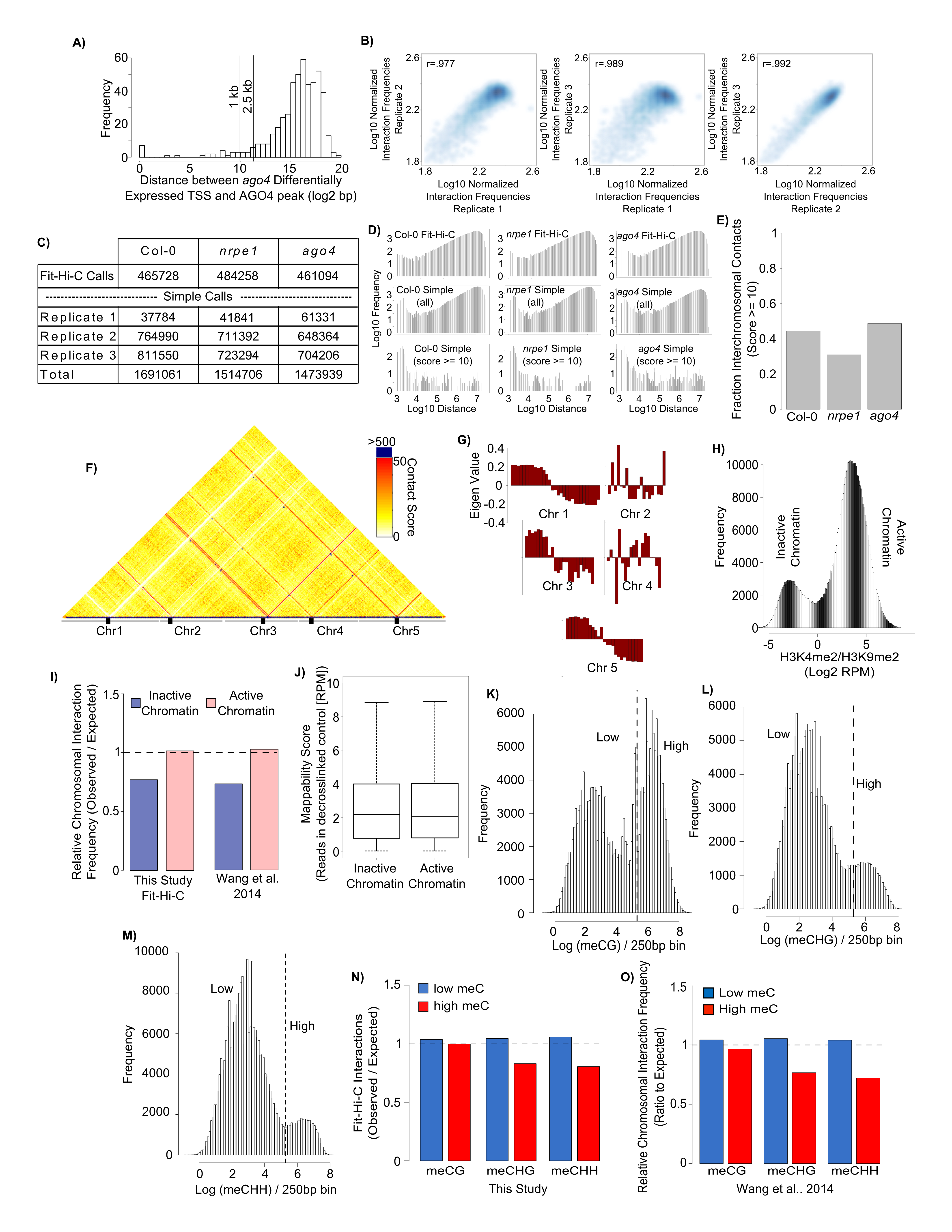

Supplement: S1 Fig — (A) AGO4 binds distant from differential genes. Histogram of distances (Log2) between TSS’s of differentially expressed genes and the closest upstream AGO4 binding site. Vertical lines indicate 1 kb and 2.5 kb putative promoter distance cutoffs. (B) Hi-C replicates correlate well. Pearson correlation (r) of individual Hi-C replicates. Plotted are contact counts between 25 kb bins normalized by the total contacts in each replicate. (C) Number of called significant interactions by Fit-Hi-C and a simpler (looser) calling method (see Methods). (D) Distances of called interactions. Histogram of significant interaction distances called by each method. (E) Inter-chromosomal interactions. Fraction of inter-chromosomal identified interactions in each genotype. (F) Contact plot of chromosomal interactions. Contact scores in 100 kb bins were calculated and plotted. Yellow to red indicates weak to strong interaction signal, blue indicates very strong interaction signal. Approximate centromere positions are noted by black squares. (G) Self-association of chromosome arms. Eigenvector plot of contact correlation along each chromosome. (H) Identification of active and inactive chromatin regions. H3K4me2 ChIP-seq signal [41] and H3K9me2 ChIP-seq signal [40] are plotted relative to each other in 250bp bins. RPM—reads per million. (I) Non-centromeric inactive chromatin is inhibited from forming chromosomal interactions. Alternate interaction calls (Fit-Hi-C) present in Col-0 from this study and interactions from Wang et al. (26) were plotted if one side was found in either inactive (blue) or active (peach) non-centromeric chromatin bins defined in Fig 1C. These were plotted as a ratio to the expected (calculated from random bins—black line). (J) Non-centromeric inactive and active chromatin have equal potential Hi-C efficiency. Boxplots represent the number of reads present in the reverse-crosslinked control from Wang et al. (26) overlapping identified inactive and active regions defi [file pgen.1006749.s001.tif]

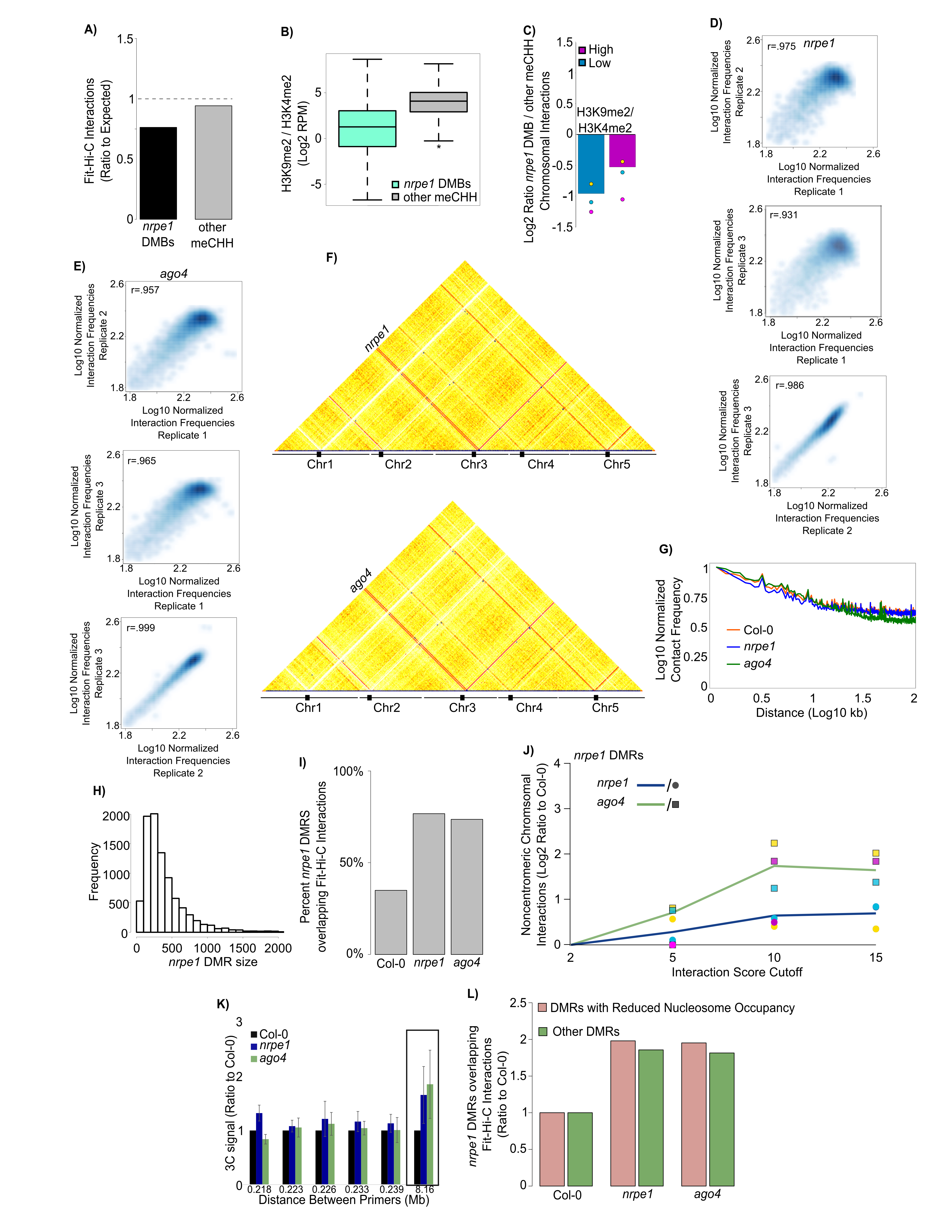

Supplement: S2 Fig — (A) Chromosomal interactions are inhibited at sites with Pol V dependent DNA methylation. Fit-Hi-C called interactions present in Col-0 were plotted if one side was found in sites with high CHH methylation as defined in S1M Fig categorized as those reduced in nrpe1 (nrpe1/Col-0 < 0.25) and those unchanged (nrpe1/Col-0 > 0.75). These were plotted as a ratio to the expected (calculated from random bins—dashed line). (B) Histone modifications at RdDM sites. Log2 ratio of H3K9me2 / H3K4me2 for nrpe1 DMBs (Differentially Methylated Bins) and other meCHH bins. (C) Interaction inhibition corresponds to RdDM. Comparison of nrpe1 DMBs to other meCHH bins with matched H3K9me2/H3K4me2 levels. Low (blue) indicates modification levels below the median nrpe1 DMB level and is applied to both bin categories. High (purple) indicates methylation levels above the median of other meCHH bins and is applied to both bin categories. Bars represent data from combined replicates while points represent individual replicates color-coded in Fig 1B. (D) Hi-C replicates in nrpe1 correlate well. Pearson correlation (r) of individual Hi-C replicates. Plotted are contact counts between 25 kb bins normalized by the total contacts in each replicate. (E) Hi-C replicates in ago4 correlate well. Pearson correlation (r) of individual Hi-C replicates. Plotted are contact counts between 25 kb bins normalized by the total contacts in each replicate. (F) Contact plot of nrpe1 and ago4 chromosomal interactions. Contact scores in 100 kb bins were calculated and plotted for nrpe1 (top) and ago4 (bottom). Yellow to red indicates weak to strong interaction signal, blue indicates very strong interaction signal. Approximate centromere positions are noted by black squares. (G) Decay curves of Col-0 Hi-C compared to nrpe1 and ago4. Reads were aligned to DpnII fragments and kept if >3 DpnII sites apart. These were assigned to 250bp genomic bins and plotted. Col-0 (orange) compared to nrpe1 Hi-C (blue) and ago4 Hi-C (gr [file pgen.1006749.s002.tif]

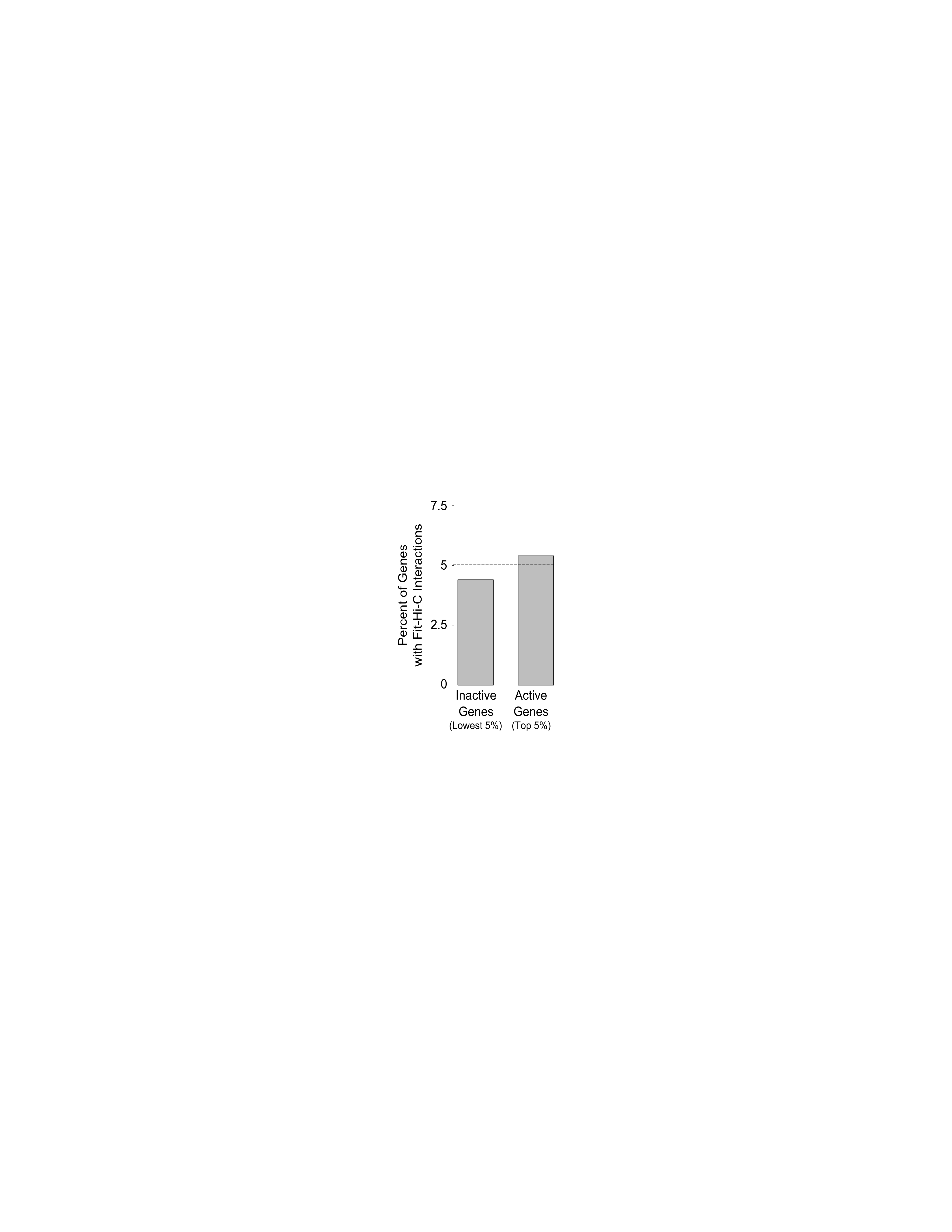

Supplement: S3 Fig — Inactive genes are less likely to engage in chromosomal interactions. The number of chromosomal interactions is plotted as a percentage of total interactions on genes. Inactive and active genes are the 5% of genes with the lowest or highest RNA-seq signals in Col-0 with promoters that are mappable in Hi-C (see Methods). Expected value from an even distribution of interactions on genes is indicated by a dashed line. (TIF) [file pgen.1006749.s003.tif]

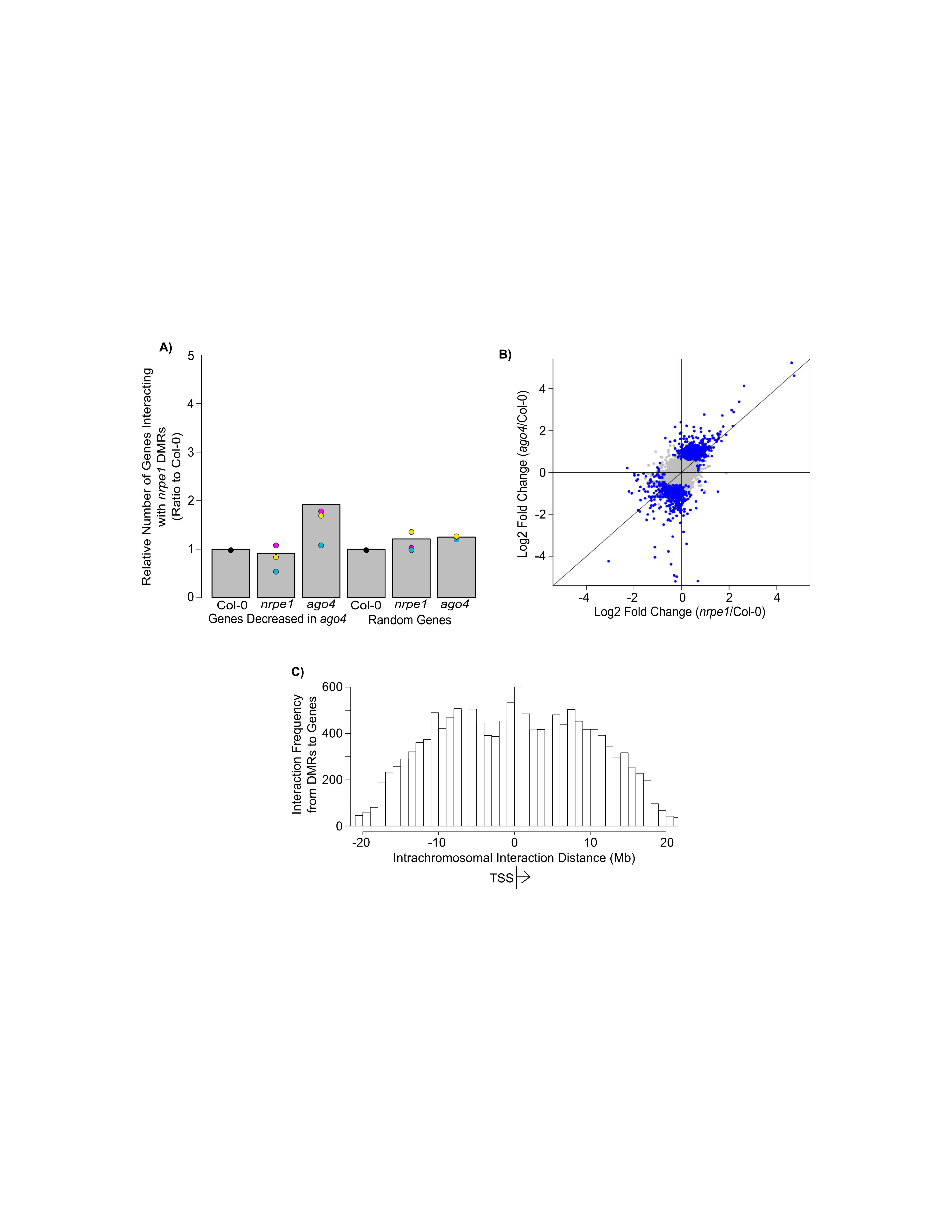

Supplement: S4 Fig — (A) Genes activated by AGO4 are slightly inhibited from interacting with nrpe1 DMRs. Plot shows numbers of genes, with decreased expression in ago4, which form chromosomal interactions to nrpe1 DMRs. Interactions are plotted in nrpe1 or ago4 as a ratio to Col-0 for genes with increased expression in ago4 and for random genes. Color coding is the same as in Fig 1B. (B) ago4 causes more drastic changes to gene expression than nrpe1. Log2 fold changes in ago4 or nrpe1 vs Col-0 from three biological repeats as calculated by EdgeR are plotted for called differential genes (blue) and total genes (grey). Diagonal line indicates a slope of 1, vertical and horizontal lines indicate no change for nrpe1 or ago4 respectively. (C) Loops between genes and DMRs are independent of distance and directionality. Chromosome loops established in ago4 are plotted if one end lies within an nrpe1 DMR and the other is in a gene promoter. Distance between ends and directionality from the transcriptional start site (TSS) is shown. (TIF) [file pgen.1006749.s004.tif]

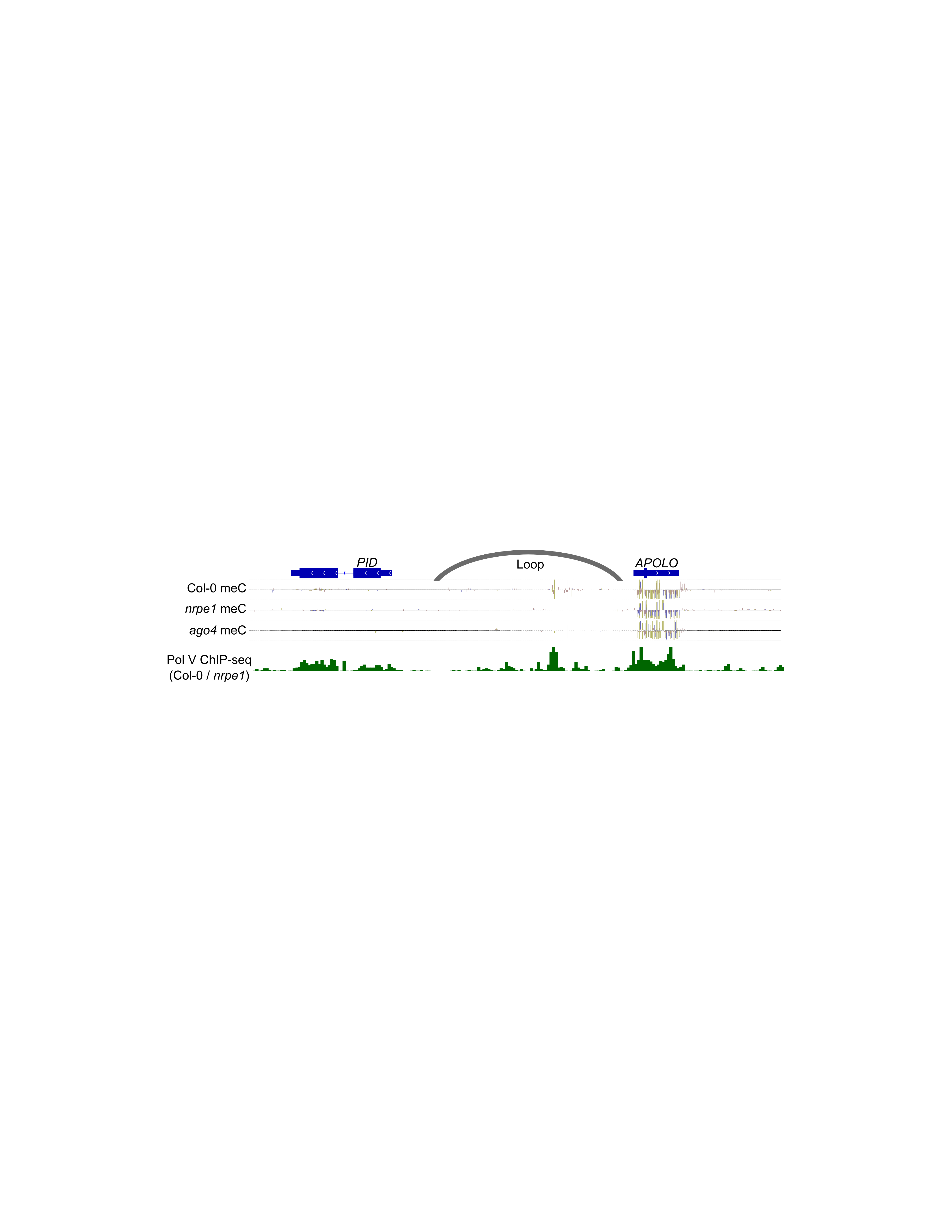

Supplement: S5 Fig — View of the published APOLO loop showing DNA methylation in Col-0, nrpe1, and ago4 [13]. meCG (yellow), meCHG (blue), and meCHH (orange) are shown. ChIP-seq signal enrichment for Pol V is also shown (green) [75]. (TIF) [file pgen.1006749.s005.tif]
